# Micro-Flow Imaging and Flow Virometry as Preferred Methods to Monitor Aggregate Formation in the Submicron and Subvisible Size Range for Two Early-Phase Vaccine Candidates

**DOI:** 10.3390/vaccines14100839

**Published:** 2026-09-23

**Authors:** Ashley Hardy, Emily Zhang, Harrison Davis, Josef Vlasak, Mingzhang Maple Wang, Malini Mukherjee, Michael J. McNevin, Lawrence Dick, Walter Wasylaschuk

**Affiliations:** 1 Pharmaceutical Analysis & Digital Technologies, Merck & Co., Inc., Rahway, NJ 07065, USA; emilyzhang@g.harvard.edu (E.Z.); josef_vlasak@merck.com (J.V.); maple.wang@merck.com (M.M.W.); malini.mukherjee@merck.com (M.M.); michael_mcnevin@merck.com (M.J.M.); lawrence_dick@merck.com (L.D.); 2 Pharmaceutical Sciences and Clinical Supply, Merck & Co., Inc., Rahway, NJ 07065, USA; davisha@udel.edu

**Keywords:** particulate matter, aggregation, dynamic light scattering, flow virometry, micro-flow imaging, human cytomegalovirus, coxsackievirus

## Abstract

Background: Unintended particle formation in vaccine samples has the potential to reduce product efficacy and impact immunogenicity. As a result, regulatory agencies routinely request tests to analyze particles in the micron size range for release. Particles may come in a variety of forms and can be inherent (e.g., from proteinaceous aggregates) or intrinsic (e.g., silicone oil from tubing or contamination from the manufacturing process) and can range in size from submicron to the visible size range. In recent years, there has been an increased interest in developing methods to detect and monitor aggregated particles as well as a desire to evaluate particle size and concentration of submicron particles in addition to the subvisible ranges routinely monitored by compendial methods. Methods: Typically utilized techniques include biophysical analytical methodologies that leverage light scattering or imaging. In this study, dynamic light scattering (DLS), micro-flow imaging (MFI), and flow virometry were evaluated as methods to detect and monitor submicron- to micron-sized particles. Results: These methods enabled assessments of stability and processability for drug substance (DS) and drug product (DP) samples of a Human Cytomegalovirus (HCMV) vaccine candidate. For this attenuated live virus vaccine candidate, MFI and flow virometry proved to be the most sensitive methods to monitor aggregate formation over the complete size range of potential aggregates. These established methods were applied to a coxsackievirus A21 vaccine candidate for process and formulation screening. Conclusions: MFI was most sensitive to changes in subvisible particle concentration of samples that were stressed via constant stirring, while flow virometry captured changes in submicron particles after stress from stirring and recirculation with a shear cell. This comprehensive screening approach combining multiple analytical methods to screen the full aggregate particle size range coupled with a broad range of forced aggregation conditions provides a framework for screening and development of vaccine formulation candidates and other modalities, such as biologics.

## 1. Introduction

Human Cytomegalovirus (HCMV), human betaherpesvirus 5, is a common Herpesvirus due to its ease of transmission and lifelong latency. Global seroprevalence is estimated at 83%, with 86% of women of childbearing age infected [1]. HCMV infection causes neurodevelopmental disabilities in infants and life-threatening diseases in immunocompromised adults, while healthy individuals are typically asymptomatic [2]. Some studies have shown that HCMV infection may also lead to cancer in healthy individuals [3]. The HCMV vaccine candidate studied here is an attenuated live virus vaccine from the AD169 strain. It is attenuated by a 15 kb deletion in the UL-b’ region of the AD169 genome. A frameshift mutation in UL131 reduces the expression of the pentameric gH complex, inhibiting infection of epithelial and endothelial cells and reducing the availability of epitopes that induce neutralizing antibodies [4]. This frameshift mutation has since been repaired, and genetic modifications have made the vaccine conditionally replication-deficient [5]. Replication in culture is dependent on the presence or absence of Shield-1, a synthetic cell-permeant ligand. The HCMV vaccine candidate antigen contains multiple viral particles, including infectious virions and non-infectious enveloped particles (NIEPs), and dense bodies [6], with sizes ranging from 150 nm to at least 400 nm. Virions are approximately 200 nm in diameter with a linear double-stranded DNA genome of approximately 236 kbp.

The handling and manufacturing of HCMV viral particle formulations may promote particle size increase or aggregation attributable to their considerable size and structural complexity. These aggregates range from the submicron to subvisible particle size and can propagate further aggregation, which may negatively impact immunogenicity and product efficacy [1]. The aggregates can also cause complications during drug substance and drug product manufacturing, such as fouling during sterile filtration; this can result in loss of material, reduced process yield, and delays in product release. Successful monitoring of particle size and aggregates allows for detection of batch-to-batch variability and can inform on process consistency. The level of aggregation required to affect immunogenicity and efficacy differs across biotherapeutics. Furthermore, mechanisms of aggregate formation are similar and well understood for some systems but remain uncertain for others [7,8].

Because of the adverse effects material aggregation can elicit, regulatory agencies require methods to monitor particulate matter in the subvisible range for release testing of injectables per ICH Q4B [9]. This requirement can present a challenge for novel vaccine development, where limited experience, scarce material, and small datasets early in development mean less knowledge about aggregate formation than will be available at the time of regulatory filing. In addition, many vaccine formulations are particulate in nature due to the presence of adjuvants or other particles contributing to efficacy, making it difficult to differentiate wanted versus unwanted particles.

Developing an understanding of product-specific aggregation requires the use of predictive and relevant forced aggregation approaches combined with appropriately discriminating analytical methods. Understanding the factors leading to product aggregation is an important consideration. There are many possible causes of protein aggregation, several of which have been identified through techniques to stress material and induce particle formation for investigation. The most common and representative methods include shaking, stirring, thermal incubation, freeze–thaw cycling, and varying pH and ionic strength [10]. Mechanical stress from shaking and stirring can cause aggregation through shear, cavitation, and interfacial effects (for example, liquid–air or liquid–liquid interfaces). Thermal incubation, particularly above the melting temperature, results in destabilization of the protein through changes in the secondary and tertiary structure, creating an environment where proteins can self-associate or become misfolded. Freeze–thaw effects depend on the rate and number of cycles, container surface properties that promote adsorption, and buffer components that may crystallize and shift pH. Changes in pH and ionic strength alter electrostatic interactions and can trigger aggregation [11]. Although the choice of forced aggregation method depends on the material and formulation properties, stirring and thermal incubation have historically proven effective for typical monoclonal antibody and vaccine products.

Several bioanalytical methods have been successfully demonstrated to monitor aggregates both qualitatively and quantitatively. In a literature study, static and dynamic light scattering analysis were combined with intrinsic fluorescence and circular dichroism to pinpoint the temperature range in which Chikungunya virus-like particles (VLPs) form molten globule-like aggregates comprising both α-helix and β-sheet conformations [12]. In another study, aggregation and particle formation in a rotavirus vaccine candidate composed of a recombinant fusion protein antigen were characterized using FTIR and SDS-PAGE, which identified shaking and freeze–thaw as the most common factors leading to particle formation [13].

In this study, the novel HCMV vaccine candidate from Merck & Co., Inc., Rahway, NJ, USA, was subjected to mechanical and thermal stress to induce aggregation and enable development of bioanalytical methods for aggregate detection. Several procedures for simulating mechanical stress, including use of a shear cell with recirculation cycles, were applied. A series of analytical characterization tools including dynamic light scattering (DLS), micro-flow imaging (MFI), and flow virometry were evaluated to detect aggregates over a broad particle size range spanning the submicron and subvisible particle size ranges. These methods enabled assessments of stability and processability for drug substance (DS), the active biological agent in bulk, and drug product (DP), the final formulated dosage form, of a HCMV vaccine candidate.

The established methods were further utilized for process and formulation screening of a coxsackievirus A21 vaccine candidate. Coxsackievirus A21 is a non-enveloped Picornavirus in the enterovirus genus. The icosahedral viral capsid is approximately 25–30 nm in diameter, made up of 60 protein subunits. Each subunit contains four viral proteins that encapsulate a single-stranded positive-sense RNA genome of approximately 7400 base pairs [14,15]. The manufacturing process for this non-enveloped vaccine system can lead to significant particle formation and aggregation. Therefore, this screening approach was essential for the preparation of a stable formulation.

## 2. Materials and Methods

### 2.1. Commercial Reagents and HCMV Samples

L-Histidine (53319-100G) and sucrose (S1888-5KG) were acquired from Sigma Life Science (Rockville, MD, USA), and 5M sodium chloride solution (AM9759) was acquired from Invitrogen (Waltham, MA, USA). Duke polystyrene standards were obtained from Thermo Scientific (Waltham, MA, USA). HCMV samples were produced internally at Merck & Co., Inc., Rahway, NJ, USA. Virus is captured from harvested viral fluid and undergoes bioburden reduction and sterile filtration. The sterile-filtered product is concentrated and diafiltrated into formulation buffer. Material is dispensed into 5L cryovault containers (Meissner Camarillo, CA, USA) and blast frozen to −70 °C. For DP formulation, DS is thawed and diluted with formulation buffer and DP stabilizer. Material is filtered through a 0.22 µm filter (Millipore, Burlington, MA, USA) prior to lyophilization.

HCMV drug substance samples were subjected to various conditions to induce aggregation, defined in this study as the presence of particles in the sample larger than the CMV virions (200 nm). Two different lots of HCMV DS were used in this study: Lot A, containing visible particulates due to the introduction of air during a filtration step, and Lot B DS as the control. HCMV DS Lot B was thawed at room temperature from −70 °C storage. Approximately 500 µL of sample was transferred from a 2 mL cryotube to a 1 mL glass HPLC vial and stirred on a magnetic stir plate for 2 and 26 h. Lot B was also diluted 100× in formulation buffer prior to subjection to thermal incubation at 37 °C for 48 h and mechanical stress by rotation at 20 rpm for 24 h. In addition to creating aggregated samples from Lot B, a sample of Lot A DS control was characterized, as it had previously shown aggregation by MFI with an increased total particle concentration of eight times more particles/mL than a typical batch. Two additional DS lots, Lots C and D, were used to conduct a thermal stability study with MFI and flow virometry methods developed from the initial study of Lots A and B.

One lot of HCMV drug product sample was subjected to similar stresses as DS to study aggregate formation. DP was reconstituted with 0.7 mL of 9.0 g/L sodium chloride. Approximately 1.4 mL of sample was transferred to a small beaker and stirred with a stir bar and stir plate for 6 and 24 h prior to analysis. DP was also vortexed for 10 and 60 s and analyzed on the same day as well as three days later. Approximately 0.7 mL of reconstituted DP was incubated in a 60 °C water bath in its original glass vial for 4 and 24 h prior to analysis. DP was also incubated in a 37 °C incubator for 3 and 7 days. In addition, DP was recirculated through a Flexicon PF6 peristaltic pump acquired from Watson Marlow (Sherborn, MA, USA) for 10 and 100 cycles at room temperature. DP was also recirculated 10, 100, and 500 times with the addition of a uniquely fabricated shear cell [16], designed specifically to generate high particle shear. The flow rate was set to 109.02 mL/min with Flexicon Accusil 1.6 mm ID tubing from Watson Marlow (Sherborn, MA, USA). Particle concentration and size were analyzed using the MFI 5200 and the Apogee A-50 Micro.

Coxsackievirus A21 drug substance was also subjected to various stress conditions to study aggregation. The neat sample went through the Watson–Marlow peristaltic pump with an in-line shear cell for 500 and 1000 recirculation cycles. Samples were also stirred with a stir bar and stir plate for 6 and 24 h and vortexed for 10 and 60 s prior to analysis.

### 2.2. DLS

The aggregated DS samples were measured by DLS using the Zetasizer Nano ZS from Malvern Instruments (Malvern, UK). DLS was used to measure particle sizes below 1 µm. The Lot B control was stirred for 2 h prior to sampling and allowed to continue stirring for up to 26 h. The 2 h and 26 h stirring timepoints were diluted 10-fold in 25 mM Histidine, 75 mM NaCl, 9% *w*/*v* sucrose, and pH 7. No further dilution was made for samples incubated at 37 °C for 48 h and rotated for 24 h. Stress conditions were measured using the Malvern Zetasizer ZS with a ZEN2112 low-volume quartz cuvette. Dispersant viscosity was set to 1.1152 cp, temperature was set to 25 °C with zero equilibration time, and the backscattering angle of detection was set to 173°. Data was processed for the intensity size distribution using the general-purpose software mode version 2.2 (Malvern Panalytical, Malvern, UK).

### 2.3. MFI

MFI, using the Micro-Flow Imaging System 5200 from Protein Simple (Minneapolis, MN, USA), was used to measure concentrations of subvisible particles larger than 2 µm and up to 70 µm in manual operation mode. Data processing morphological filters were set to exclude particles with circularity > 0.9, which is consistent with particles that are circular in shape, such as bubbles and silicone oil. The Lot B control, stirred for 2 and 26 h, was diluted 10-fold in the DS buffer formulation (25 mM Histidine, 75 mM NaCl, 9% *w*/*v* sucrose, pH 7 buffer) to a target concentration between 10,000 and 900,000 particles/mL. No further dilution was made for samples incubated at 37 °C for 48 h and rotated for 24 h. The instrument was flushed with formulation buffer in between each measurement. Each DP condition was prepared at a 10-fold dilution in the DS buffer and measured by MFI with the Bot1 autosampler. All data processing morphological filters were removed for DP analysis and for DS thermal stability studies to allow collection of all particles > 1 µm in size. Cocksackievirus A21 samples were measured neat with the same instrument parameters used for CMV DP. The instrument was flushed with formulation buffer (10 mM Histidine, 10 mM Tris, 150 mM NaCl, 5% sucrose, 0.005% PS80, pH 7.2) in between each measurement.

### 2.4. Flow Virometry

The Apogee Flow Cytometer A-50 Micro from Apogee Flow Systems (Hertfordshire, UK) was used to compare particle size and concentration of submicron particles larger than CMV in aggregated samples to the Lot B control. The Lot B control, stirred for 2 and 26 h, was diluted in 25 mM histidine, 75 mM NaCl, 9% *w*/*v* sucrose, and pH 7. To measure the virus peak in the Apogee histogram, samples were diluted 2000-fold to a target concentration of 100,000–200,000 events/µL and diluted 30-fold for aggregate measurements. No further dilution was made for aggregate measurement of samples incubated at 37 °C for 48 h and rotated for 24 h. The 405 nm side-scatter threshold was set to 40 to measure virus primary particles and was adjusted to 10,000 to measure aggregates (and to exclude measurement of dense bodies). The flow rate was set to 3.01 µL/min for a sample volume of 130 µL and a laser power of 70 mW. Each DP condition, with the exception of the 10 and 100 peristaltic pump recirculation cycles with the shear cell, was diluted 200-fold in the formulation buffer for virus particle measurement and a 30-fold dilution in the formulation buffer for the aggregate measurement. The aggregate threshold for DP measurements was set to measure particles > 750 nm, which was set via measurement of a 750 nm silica size standard (147084-10) from Corpuscular (Cold Spring, NY, USA). For coxsackievirus A21, samples were measured neat with the flow rate set to 1.50 µL/min for a sample volume of 130 µL, a laser power of 300 mW, and a 405 nm side-scatter threshold set to 100 to measure aggregate particle concentration.

## 3. Results

### 3.1. Assessment of DLS, MFI, and Flow Virometry Method Capability for Detecting Aggregation

The selection of an appropriate method to measure particle size and concentration is based on several criteria including the size and properties of the primary particles and the aggregates, as well as the robustness of the measurement system to detect these primary particles and aggregates. In this case, the HCMV antigen primary particle size is known to be approximately 200 nm, with aggregates possible in both the submicron and subvisible particle size range. Given the diversity of aggregation mechanisms and aggregate properties, a series of forced stress conditions to simulate thermal and mechanical stress were applied in an attempt to produce representative samples with aggregate profiles with which various methods could be evaluated. For this product, we evaluated DLS, which can measure particle size in the submicron and subvisible ranges, flow virometry for detection of submicron particles, and MFI for detection of subvisible particles.

HCMV drug substance was subjected to various stressors to induce aggregation as described in Section 2.1. Lots A and B are two different batches from the same process. Lot A contains visible particulates induced by unoptimized downstream processes, and Lot B is the control from the optimized process. The stress conditions include mechanical (rotation, stirring) and thermal stress (37 °C). Samples were analyzed by DLS, MFI, and flow virometry.

The average particle size measured from DLS analysis exhibited a minor increase between the aggregated samples and the Lot B control sample, as seen in Figure 1A. The Lot B control showed an average size of 247 nm. Lot A, which contained visible aggregates, showed an increase of 30 nm in comparison, but only one primary peak was observed in Figure 1B. A larger increase in z-average as well as a shift in the size distribution was expected in Lot A, which is known to contain additional micron-sized particles. Evidence of multiple populations is indicated by the increase in polydispersity of Lot A (Figure 1C) but does not reveal additional information about the exact size of the larger particles. Similar trends were observed in stressed Lot B, with the z-average (Figure 1A) size in the 200 nm range and an increase in polydispersity (Figure 1C) across stress conditions (see Figure 1). The lack of sensitivity for detecting particles in the micron-size range by DLS rendered the method unsuitable as the primary aggregation method required for routine testing of clinical material to support regulatory filings. This finding highlighted the importance of supplementing DLS measurements with orthogonal analytical techniques, as DLS may underestimate the formation of aggregates.

A small increase in particle concentration for aggregated DS Lot B conditions was observed when measured by MFI (see Figure 2A). The greatest increase occurred after stirring for both 2 h and 26 h, with a 2-fold increase in particle concentration. Lot A showed an 8-fold increase in particle concentration in comparison to the Lot B control. This increase in micron-sized particles correlated with visual observations of aggregates in Lot A. A 3-fold and 2-fold decrease in particle concentration was observed in Lot B after 37 °C incubation for 48 h and rotating for 24 h, respectively. Unlike DLS, which is unable to provide information on particles in the micron-size range, MFI was capable of displaying the significant differences in particle concentration (1–70 µm) between the Lot B control and Lot A.

Initial assessment of flow virometry with DS samples proved that the technique had great sensitivity for differentiating aggregated and control samples, as shown in Figure 3. Flow virometry is capable of detecting particles in the size range spanning the submicron (as small as approximately 70–100 nm) to micron-sized particles (based on analysis of polystyrene bead standards). Lot A, with visible particulates, showed significantly higher total particle concentration, with a 16-fold increase compared to the Lot B control in Figure 2B. The greatest increase in particle concentration, when stressing Lot B, was observed when incubating at 37 °C for 48 h, resulting in a 16-fold increase, followed by stirring for 26 h with a 7-fold increase and rotating for 24 h with a 5-fold increase in particle concentration. A smaller, 2-fold increase in particle concentration was observed in samples stirred for 2 h. In addition to an increase in particle concentration, a shift in the size distribution towards larger particles is seen in the flow virometry histogram (Figure 2C) from stirring over time. Light scattering increases for larger particles in the stirred sample, marked by an increased sharp peak on the right side of the distribution.

### 3.2. Monitoring Aggregate Formation in HCMV Drug Product via MFI and Flow Virometry

HCMV drug product was also subjected to various stressors to induce aggregation as described in Section 2.1. Following lessons learned from the DS aggregation studies, MFI was selected to measure subvisible particles and flow virometry for submicron to micron particles for analysis of the DP samples. These techniques were used to assess the impact on particle size from lyophilization as well as simulated stressors, including thermal stress (37 °C, 60 °C) and mechanical stress (stirring, vortexing, and different degrees of pumping and shearing).

In HCMV DP, an increase in total particle concentration, in comparison to the DP control, was observed for stressed samples when measured by MFI, as shown in Figure 4A–C. Incubation at 60 °C resulted in increased particle concentration for particles larger than 5 µm, with a 2-fold increase after four hours of incubation and a 22-fold increase after one day. Vortexing also had a great impact on particle concentration, with a 5-fold increase in subvisible particles after 60 s followed by measurement immediately after vortexing. The measurements of subvisible particles 3 days after 60 s of vortexing showed a 10-fold increase and indicate that the shear stress of vortexing may seed additional aggregate formation. Pumping samples with a peristaltic pump had lesser effects on subvisible particle concentration with and without the shear cell, as well as stirring for up to one day.

An increase in % aggregates (expressed as a number %), for particles greater than 750 nm, was observed for most stressed DP samples measured by flow virometry (see Figure 5A–C). The greatest increases resulted from stirring for 24 h followed by vortexing for various time intervals and pumping with a shear cell after 500 recirculation cycles. Increases in particle concentration, although not as severe, were observed with incubation at 37 °C and 60 °C for 24 h. It was noted that with vortexing, particle concentration was higher after analyzing the sample three days post-preparation as opposed to analyzing on the same day. Particle concentration measured by flow virometry was not affected by pumping with increasing recirculation without the shear cell. A trend in decreasing total particle concentration was also observed in aggregated DP samples (see Figure 6). This decrease in total concentration and increase in % aggregates was consistent with the shift in size distribution observed in aggregated DS samples (see Figure 2C).

Mechanical stress with extended stirring of Lot B DS had the greatest impact on subvisible aggregates, as measured using MFI, while submicron particle counts by flow cytometry increased more after thermal incubation at 37 °C. Stirring for 26 h resulted in increased aggregate formation across the full particle size range. Incubation at 37 °C and rotating had the least impact on subvisible particles, while stirring for 2 h had little effect on submicron particles (see Figure 3). For DP, the highest increase in particle counts in the subvisible range resulted from thermal stress. Mechanical stress generated more aggregates in the submicron range. Vortexing for 60 s and thermal incubation at 60 °C drove aggregation in both the submicron and subvisible range. Subvisible particles in DP were least affected by pumping with and without the shear cell, stirring, and vortexing for 10 s. Particles in the submicron range were also not impacted by pumping without the shear cell (see Figure 7). Increasing the duration of stress conditions resulted in increased aggregate formation for both the DS and DP samples.

### 3.3. Monitoring Aggregate Formation During Thermal Stability in HCMV Drug Substance via MFI and Flow Virometry

Having demonstrated that the MFI and flow virometry methods were sensitive to different types of forced stress and able to detect product aggregation, the performance characteristics of the MFI and flow virometry methods were verified through a phase-appropriate validation. The MFI method was found to meet all criteria for accuracy, repeatability, precision, range, linearity, and specificity. Most notable was that the intermediate precision showed a 4% RSD for DS and 12% RSD for DP. Repeatability (six measurements by two analysts over 2 days) was found to be 4% RSD for DS and 1% RSD for DP. The flow virometry method was found to meet all criteria for accuracy, repeatability, precision, range, linearity, and specificity for measurements of the primary antigen peak and for % aggregates. For the measurement of the primary antigen peak, the repeatability (six measurements by one analyst) showed an 18% RSD for DS and a 8% RSD for DP. Repeatability for measurements of % aggregates was found to be 34% RSD for DS and 8% RSD for DP. Intermediate precision for the primary antigen peak was 2% for DS and 10% for DP. Intermediate precision of % aggregates for DS was 17% and 5% for DP.

These verified MFI and flow virometry methods were applied to monitor aggregates for an HCMV drug substance thermal stability study. Two different drug substance lots (Lot B and Lot C) were staged on stability and characterized using flow virometry and MFI in Figure 8 and Figure 9, respectively. The drug substance lots were analyzed at time zero on the day of the stability study start. For Lot C, a portion of the drug substance was aliquoted into matrix tubes and placed directly in −70 °C and 5 °C CTUs, while the remaining volume was blast frozen and stored at −70 °C. Samples from each storage condition were measured by MFI and flow virometry at numerous intervals post-storage, from 1 day to 4 months for Lot C and 6 months for Lot D.

Particle concentration by MFI for Lot C remained steady at 2–8 °C up to 4 months. A 5-fold increase post-blast freezing was observed, followed by steady particle concentration post-storage at −70 °C. Shelf-frozen material, stored at −70 °C, had a gradual increase in particle concentration over time, with a 5-fold increase compared to T0. For Lot D, a 2-fold increase was observed in particle concentration over 6 months at 2–8 °C. Shelf-frozen material at −70 °C showed a steady increase in particle concentration, with a 5-fold increase at 6 months.

Flow virometry measurement of Lot D showed a 3-fold increase in % aggregates at 2–8 °C. A 5-fold increase was observed post-blast freezing, with up to a 10-fold increase with storage at −70 °C post-blast freezing. Shelf freezing at −70 °C showed a steady increase in % aggregates with a 6-fold increase over time. Storage temperature had a significant impact on % aggregates for Lot D, with a 51-fold increase over 6 months of storage at 2–8 °C. Only a 2-fold increase was observed when stored at −70 °C. Figure 10 demonstrates that Lot C was more sensitive to freezing, with an increase in particles in both the submicron and subvisible range, while Lot D was more sensitive at 2–8 °C storage, with an increase primarily in submicron particles. This provided valuable insight and guided decisions for formulation and process parameter selection.

### 3.4. Monitoring Aggregate Formation in Coxsackievirus A21 DS via MFI and Flow Virometry

The HCMV screening approach was also applied to a different type of vaccine candidate, coxsackievirus A21. Coxsackievirus A21 is a non-enveloped Picornavirus in the enterovirus genus. The icosahedral viral capsid is approximately 25–30 nm in diameter, and it is made up of 60 protein subunits. Each subunit contains four viral proteins that encapsulate a single-stranded positive-sense RNA genome of approximately 7400 base pairs [14,15]. Coxsackievirus A21 is prone to aggregation as it is non-enveloped. Therefore, the focus of the screening study was to evaluate the physical force sensitivity of the drug substance. Particle concentration was monitored in coxsackievirus A21 DS when subjected to various stressors, including recirculation with a shear cell, stirring, and vortexing, as shown in Figure 11. An increase in total particle concentration was observed in all stress conditions when compared to the control. The most significant increase in particle concentration was observed after stirring for 24 h. Total particle concentration of these samples was also measured by flow virometry. An increase in particle concentration was observed in all stressed conditions with the exception of stirring for 1 h and vortexing for 10 s. The greatest increase was observed with recirculation with the shear cell and stirring for 24 h. Stirring and vortexing for an extended period of time, as well as subjecting to 1000 recirculation cycles, had the greatest impact in the subvisible range, while stirring and recirculation triggered a significant increase in the submicron range (see Figure 11C).

## 4. Discussion

Currently, there is no compendial method to monitor aggregates in vaccine products due to the diversity of vaccine technology and properties. In the literature studies, size-exclusion chromatography (SEC) [17], nanoparticle tracking analysis (NTA) [18], and dynamic light scattering (DLS) [19] have been used to monitor particle size of aggregates, while micro-flow imaging (MFI) [20] has previously been established as a useful technique to examine particle size as well as morphology of aggregates. Transmission electron microscopy (TEM) [21] has been used to differentiate aggregates in vaccine samples, and flow cytometry (coupled with fluorescence immunostaining) [22] has been used to identify individual viral particles from the bulk sample. In addition, sodium dodecyl sulfate–polyacrylamide gel electrophoresis (SDS-PAGE) and Western blot have been used to examine particle protein composition [23].

Although SEC, NTA, and DLS are commonly used to detect and monitor particle size of aggregates, each technique has some limitations. Electrostatic and hydrophobic interactions with the stationary phase often lead to low recovery of protein aggregates using SEC [13,24]. Due to decreased Brownian motion of larger particles, NTA and DLS cannot accurately measure size for particles greater than 1 µm [25]. In addition, DLS is not suitable for polydisperse HCMV samples as the technique is performed in batch mode, outputting an average size in which the impact of low levels of large aggregates can be masked by the dominant light scattering intensity of the primary particles (see SI). This study demonstrated that use of MFI and flow virometry for aggregate analysis in vaccines allows for enhanced sensitivity for detection and measurement of aggregates over the submicron to subvisible size range, which is historically a gap in routine particle characterization that has been previously documented [26]. It was noted that changes in particle concentration were more apparent in flow virometry measurements than MFI. This can be attributed to the size ranges in which each instrument is capable of measuring. MFI measures particles only in the micron size (>1 µm), while flow virometry covers the submicron particle size range (~70 nm and greater). The Apogee flow cytometer is, therefore, able to capture more particle counts over a wider particle size range and has the advantage of being able to monitor the primary antigen particles as well as aggregates for vaccines, such as HCMV.

The combination of these two methods provided comprehensive particle size information over the broadest size range, which has notably been a gap in conventional particle characterization. Evaluating these methods simultaneously enabled monitoring of particle formation in the entire size range of the CMV vaccine candidate. Monitoring particle formation in the submicron range provides insight into potential particle growth from the submicron to subvisible range, while studying particle growth in the submicron range can provide early indicators for potential formation of visible particulates. In addition to the wide particle size range provided by flow virometry, fluorescent channels allow for identification of proteinaceous aggregates with fluorescent staining, such as PROTEOSTAT or Alexa Fluor 488. New models also come equipped with a plate holder to enable higher throughput sample analysis. Semi-high throughput sample analysis is also a benefit for MFI with the addition of the Bot 1 autosampler. In addition, MFI provides images (see Figure S1) of individual particles in the sample that can be used for additional analysis, such as predictive modeling with machine learning [27].

The DLS instrument’s inability to significantly detect aggregation in HCMV samples prompted a study of polystyrene beads for further understanding (see SI). This study revealed that the impact of aggregates on particle size measured by DLS is highly dependent on the ratio of larger particles to smaller particles. As the particle concentration of large aggregates in a sample increases relative to the smaller particles, the presence of these aggregates becomes increasingly apparent in the Z-average (Table S1 and Figure S2). In contrast, large, obvious increases in particle concentration for stressed samples were observed through MFI and flow virometry, which drove the decision to employ these methods to detect aggregation in the HCMV vaccine candidate.

Forced stress conditions were applied in this study to mimic real-world conditions. For DS, rotating, stirring with a stir bar, and thermal incubation were implemented. Stirring was more prone to produce the formation of subvisible particles, while thermal incubation at 37 °C was more effective in forming submicron particles. For DP, stirring with a stir bar, vortexing, and thermal incubation at 37 °C and 60 °C were used to generate aggregates. In addition to these traditional mechanisms for aggregation formation, this study also used a shear cell coupled with recirculation via a peristaltic pump. Vortexing for 60 s and thermal incubation at 60 °C generated more aggregates in the subvisible range. In the submicron range, stirring, recirculation with the shear cell, and thermal incubation at 60 °C were most informative. Vortexing for 60 s showed a higher increase in subvisible particles in comparison to stirring for 24 h. The opposite was observed in the submicron range. This indicates that the stirring conditions applied created small submicron aggregates while vortexing created larger subvisible aggregates in DP.

The quantity and size of particles formed through stress conditions is highly dependent on the type of stress applied and the properties of the antigen or molecule being studied. For HCMV, shear stress and thermal incubation proved to have the biggest impact on particle formation for both DS and DP. Particle formation from stirring depends on the stir speed and time, sample volume, and protein concentration. Contact with the stir bar results in absorbed protein on the surface, promoting structural deformation, which leads to additional protein absorption and agglomeration. These agglomerates are then released into solution through contact between the stir bar and container [28]. The result is a loss of primary particles with an increase in particle aggregates, which can be detected in the submicron and subvisible particle size ranges. Vortexing creates an environment favorable for self-association via an increase in the air–water interface. With thermal incubation at 37 °C, an increase was observed in the submicron range for DS, while increasing the incubation temperature to 60 °C, well above the HCMV melting temperature of approximately 40 °C, resulted in more particle formation in subvisible particles for DP. This comprehensive aggregate particle screening, spanning the submicron and subvisible size ranges, coupled with comprehensive forced aggregation studies, can provide valuable guidance for the DS and DP manufacturing process and formulation design studies and impact analytical sample preparation best practices during routine analytical analysis.

A similar approach for inducing aggregation, followed by analysis with MFI and flow virometry, was applied to a coxsackievirus A21 vaccine candidate for process and formulation screening. A significant increase in particle concentration in the subvisible range was observed by MFI post-stirring for 24 h. Stirring and recirculation with the shear cell had the greatest impact on particle formation in the submicron size range. It was noted that different trends in particle formation were observed when comparing coxsackievirus A21 to HCMV. This was primarily attributed to the differences in structure of the two antigens, as coxsackievirus A21 is non-enveloped and more susceptible to stress-induced aggregation.

## 5. Conclusions

This study explored common characterization techniques, coupled with flow virometry applied in a non-traditional way, to assess the ability to detect and monitor aggregates covering the submicron and subvisible size ranges, for two different vaccine candidates. Stressed HCMV samples were evaluated by DLS, flow virometry, and MFI for suitability in detecting and quantitating aggregate formation. This study raises awareness of the limitations, when applied to this HCMV vaccine candidate, of one of the most utilized techniques for particle size and aggregate characterization, dynamic light scattering. MFI and flow virometry proved to be most sensitive for detecting aggregation for this particular vaccine candidate. The flow virometry measurement provided insight into particle size and aggregates in the submicron range, enabling monitoring of the primary viral particles in the 200 nm range as well as aggregates forming in the upper limits of the submicron range, whereas the MFI results provided complementary information for particle formation in the subvisible range. Forced stress conditions were applied in this study to mimic manufacturing and handling conditions. Stirring and vortexing are typically applied to samples during analytical testing and will also mimic the shear effects experienced by DS or DP during processing. Samples may be affected by thermal instability during shipment due to temperature excursions or insufficient temperature control during transport through different climatic zones [29]. Recirculation was introduced, with the use of a novel shear cell, as a model for stress endured during batch processing, such as buffer exchange via tangential flow filtration. For drug products, it was determined that both stirring and vortexing lead to a significant decrease in total submicron primary particles, but these produce a different population of aggregates. For DP stirred for 24 h, an approximate 30% decrease in submicron particles was associated with an increase in submicron aggregates (>750 nm) and a decrease in subvisible particles, whereas after 60 s of vortexing, an approximate 30–50% decrease in submicron particles was associated with a significant increase in subvisible particles. A similar forced aggregation procedure was applied to the coxsackievirus A21 drug substance. This material was found to be more sensitive to recirculation than HCMV.

In addition, the formation of submicron aggregates was very common for the coxsackievirus A21 DS, where stirring led to an increase in submicron particles as measured by flow virometry and subvisible particles as measured by MFI. These types of trends emphasize the need for comprehensive screening of submicron and subvisible particles for a complete assessment of product sensitivity and propensity to aggregate. This comprehensive screening approach, combining multiple analytical methods coupled with a broad range of forced aggregation conditions, provides a framework for screening and development of vaccine formulations. The analytical strategy outlined here can be applied to other modalities, such as biologics, as techniques used to characterize monoclonal antibodies often overlap with vaccines. Future studies to monitor aggregation in vaccines may include static light scattering for enhanced resolution in polydisperse aggregated samples, holographic microscopy to distinguish aggregates greater than 500 nm based on size and refractive index, and fluorescence immunostaining coupled with flow virometry to positively identify aggregate composition.

## Figures and Tables

**Figure 1:**
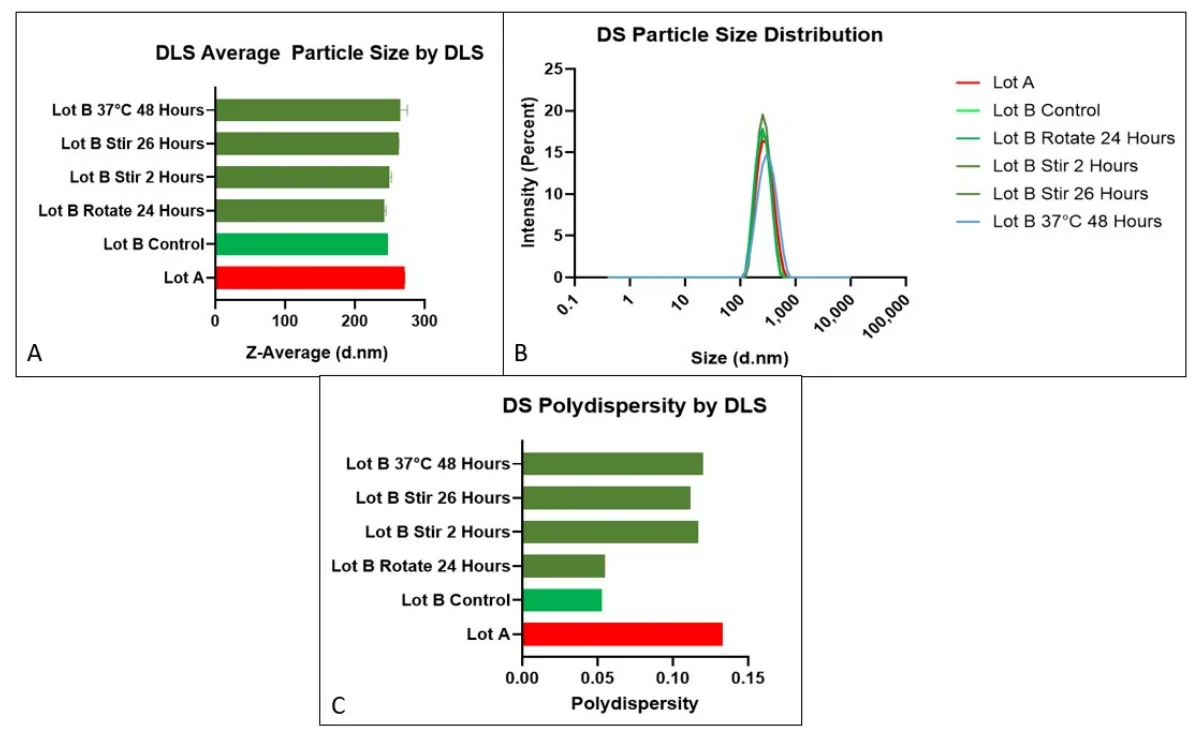
DLS analysis of DS lots. Lots A and B are two different batches from the same process. Lot A contains visible particulates, and Lot B is the control, which was subjected to mechanical stress by stirring with a stir bar for 2 h and 26 h, rotating for 24 h, and holding at 37 °C for 48 h. Samples were prepared and measured with *n* = 3. (**A**) Comparison of average particle size. (**B**). Overlay of particle size distribution. (**C**) Comparison of polydispersity.

**Figure 2:**
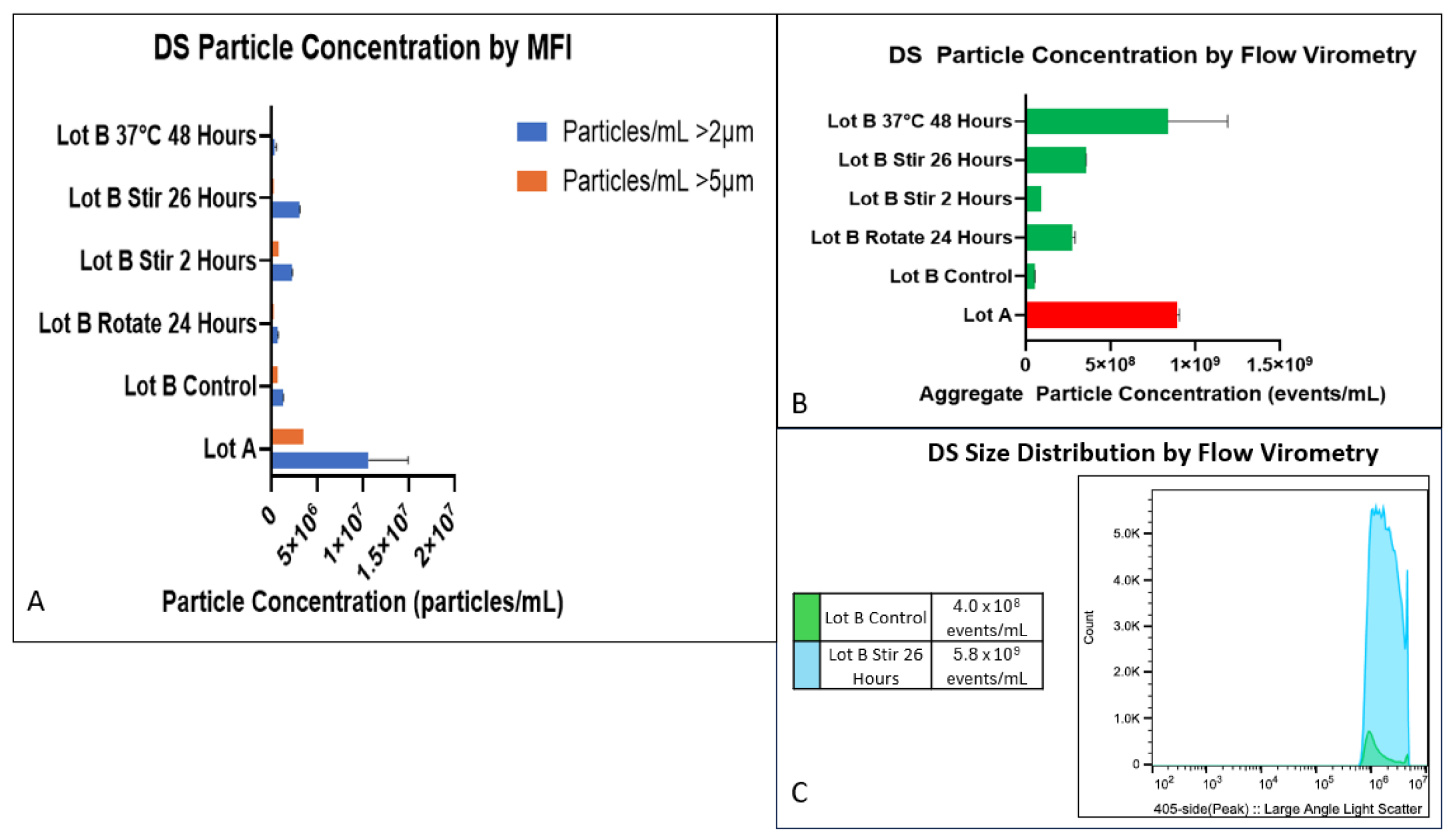
Particles in stressed Lot B DS characterized by MFI and flow virometry. (**A**) MFI of DS lots reporting particles > 2 µm and 5 µm. (**B**) Flow virometry of DS lots reporting aggregate particles. (**C**) Size distribution of overlay of the Lot B control and Lot B stirred for 26 h. Samples prepared and measured with *n* = 3 except for stirring for 2 and 26 h, which were *n* = 2.

**Figure 3:**
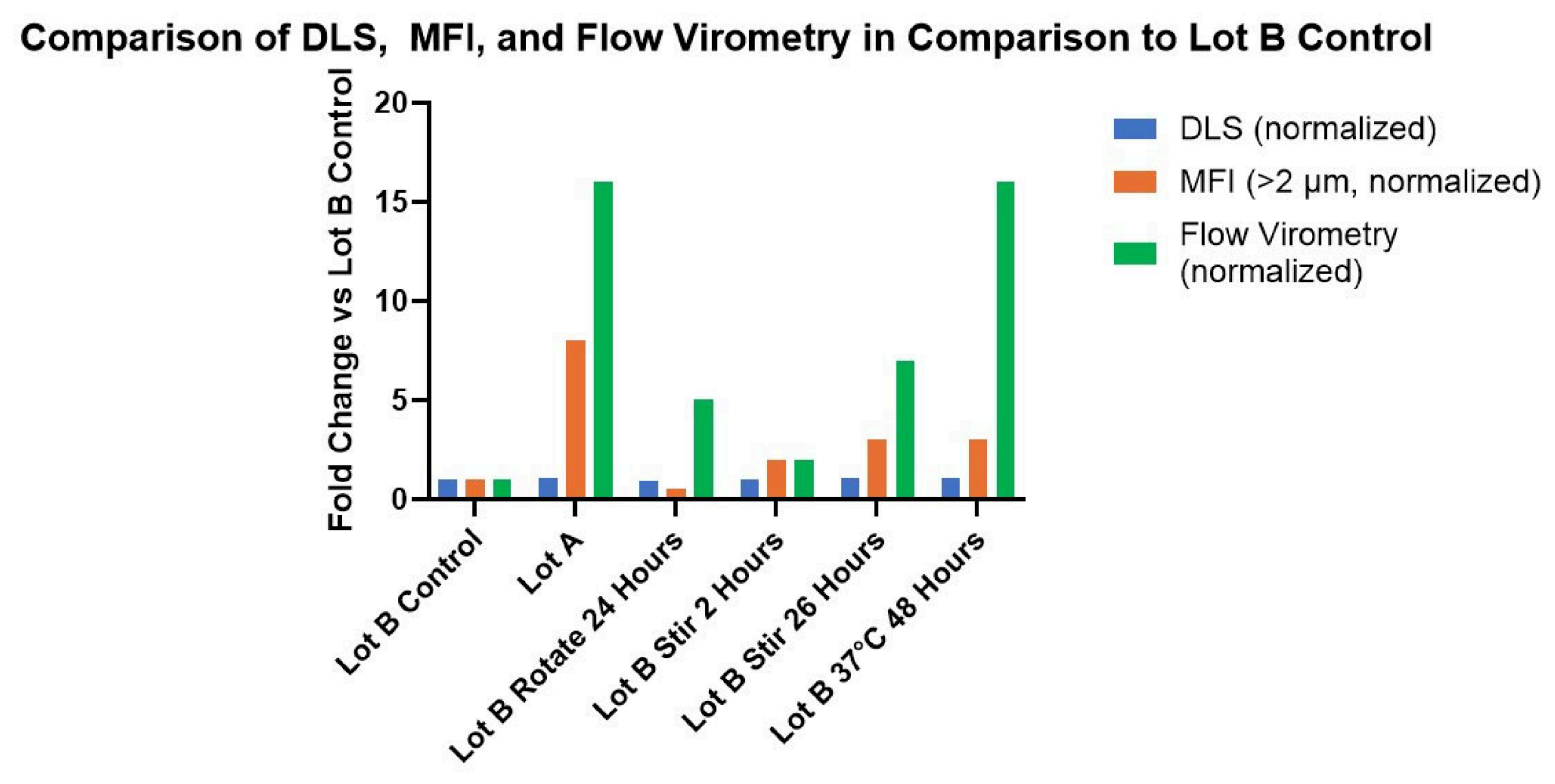
Summary of the results for DS measurements by DLS, MFI, and flow virometry. Comparison of DLS, MFI, and flow virometry results for DS. The data is normalized to the Lot B control results.

**Figure 4:**
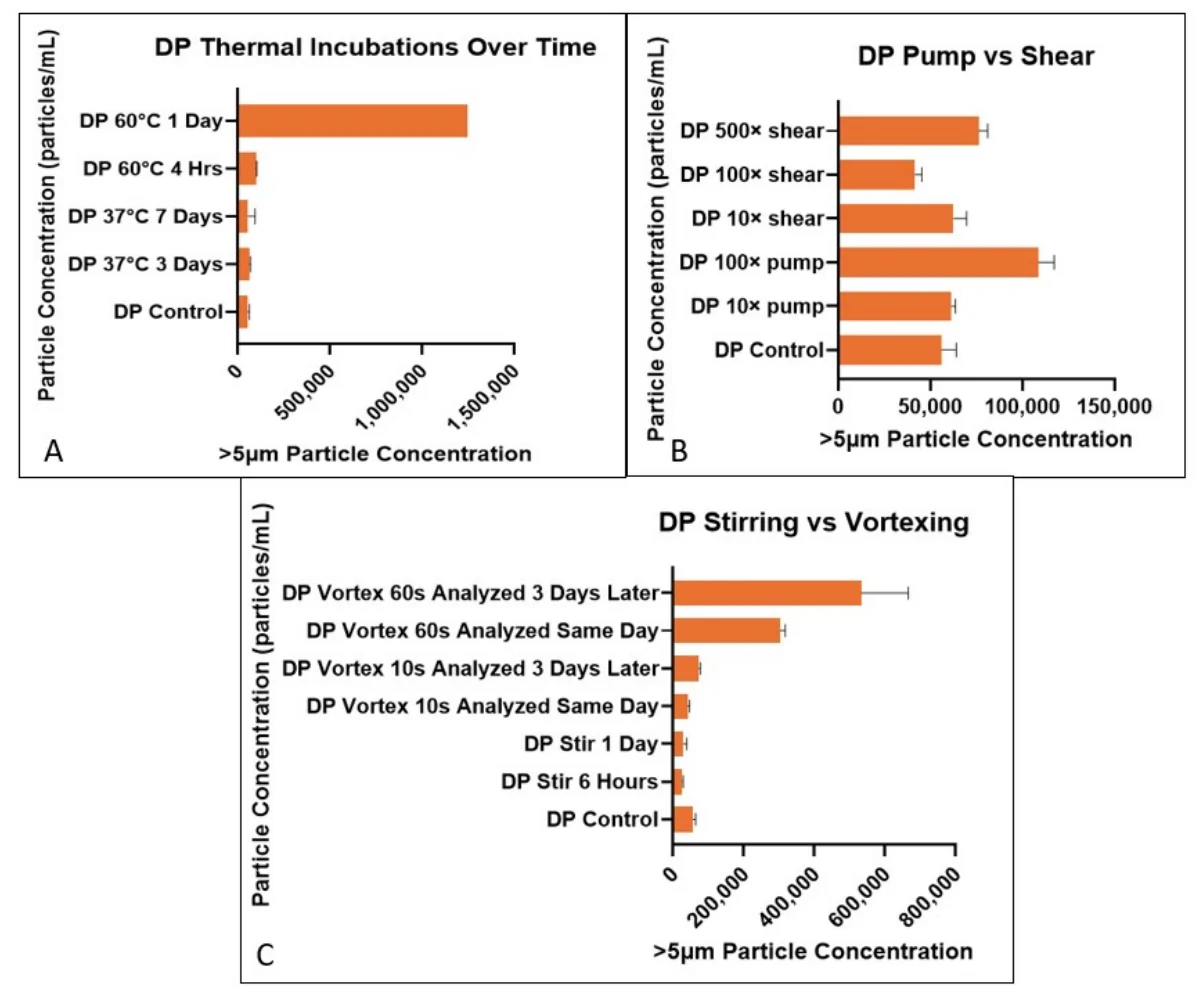
MFI analysis of DP. DP was subjected to a series of stress conditions. (**A**) Thermal incubation over time at 37 °C and 60 °C. (**B**) Recirculation with a peristaltic pump and shear with a shear cell. (**C**) Stirring compared to vortexing. Samples were prepared and measured with *n* = 2. Due to limited sample volume, DP incubated at 60 °C for one day was prepared and measured with *n* = 1.

**Figure 5:**
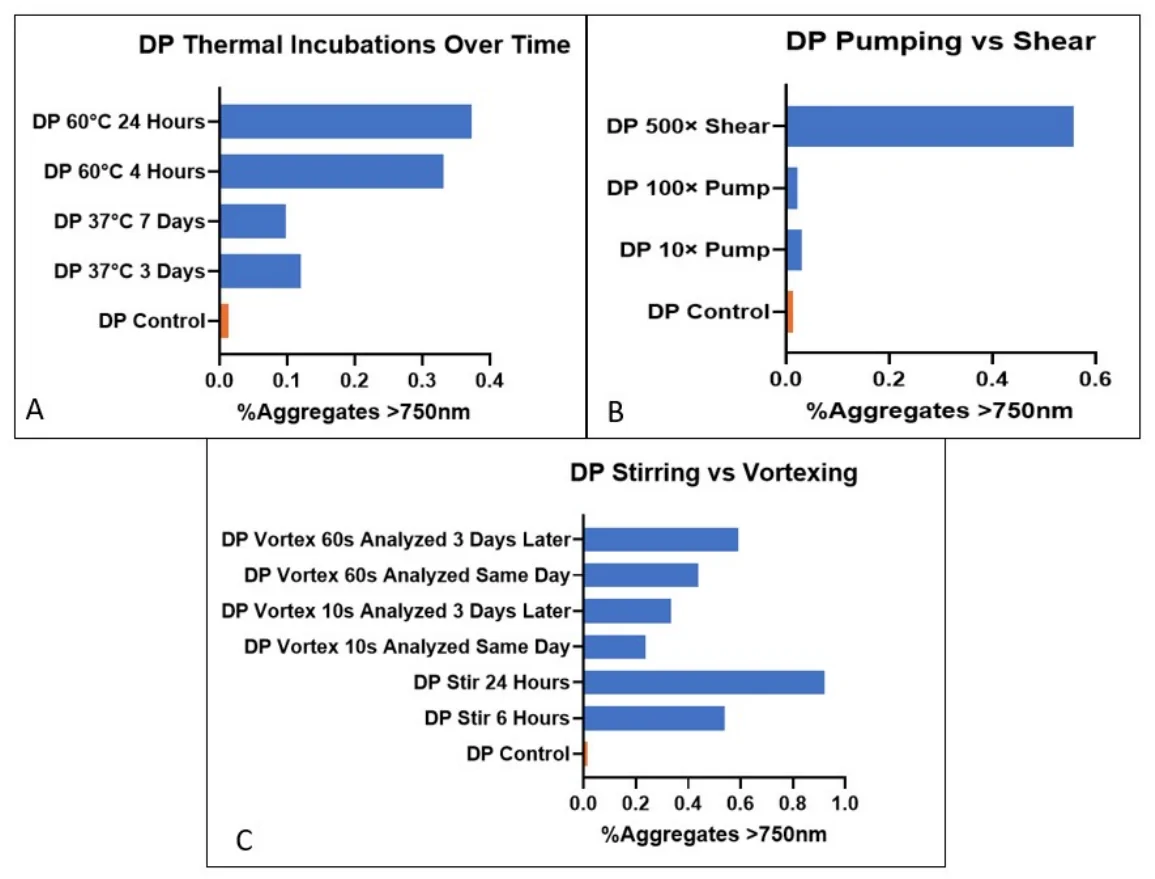
Flow virometry of DP % aggregates > 750 nm. (**A**) Thermal incubations over time. (**B**) Pumping with a peristaltic pump compared to shear with a shear cell. (**C**) Stirring compared to vortexing. Due to limited sample volume, samples were prepared and measured at the aggregate threshold with *n* = 1.

**Figure 6:**
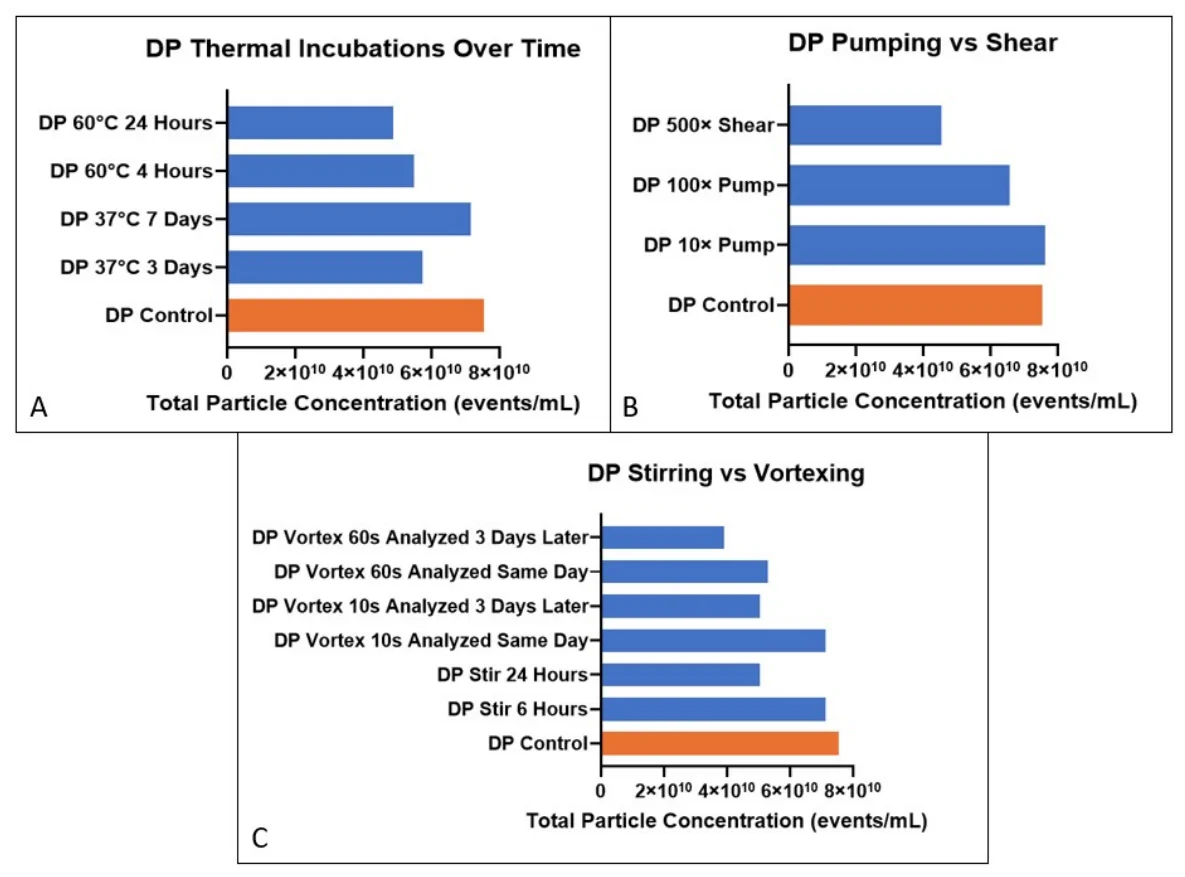
Flow virometry of DP; total particle concentration. (**A**) Thermal incubation of DP at 37 °C and 60 °C. (**B**) Pumping with a peristaltic pump compared to shear with a shear cell. (**C**) Stirring compared to vortexing. Samples were prepared and measured with *n* = 2 for DP control, pumping, and shear. Due to limited sample volume, all other stress conditions were prepared and measured with *n* = 1.

**Figure 7:**
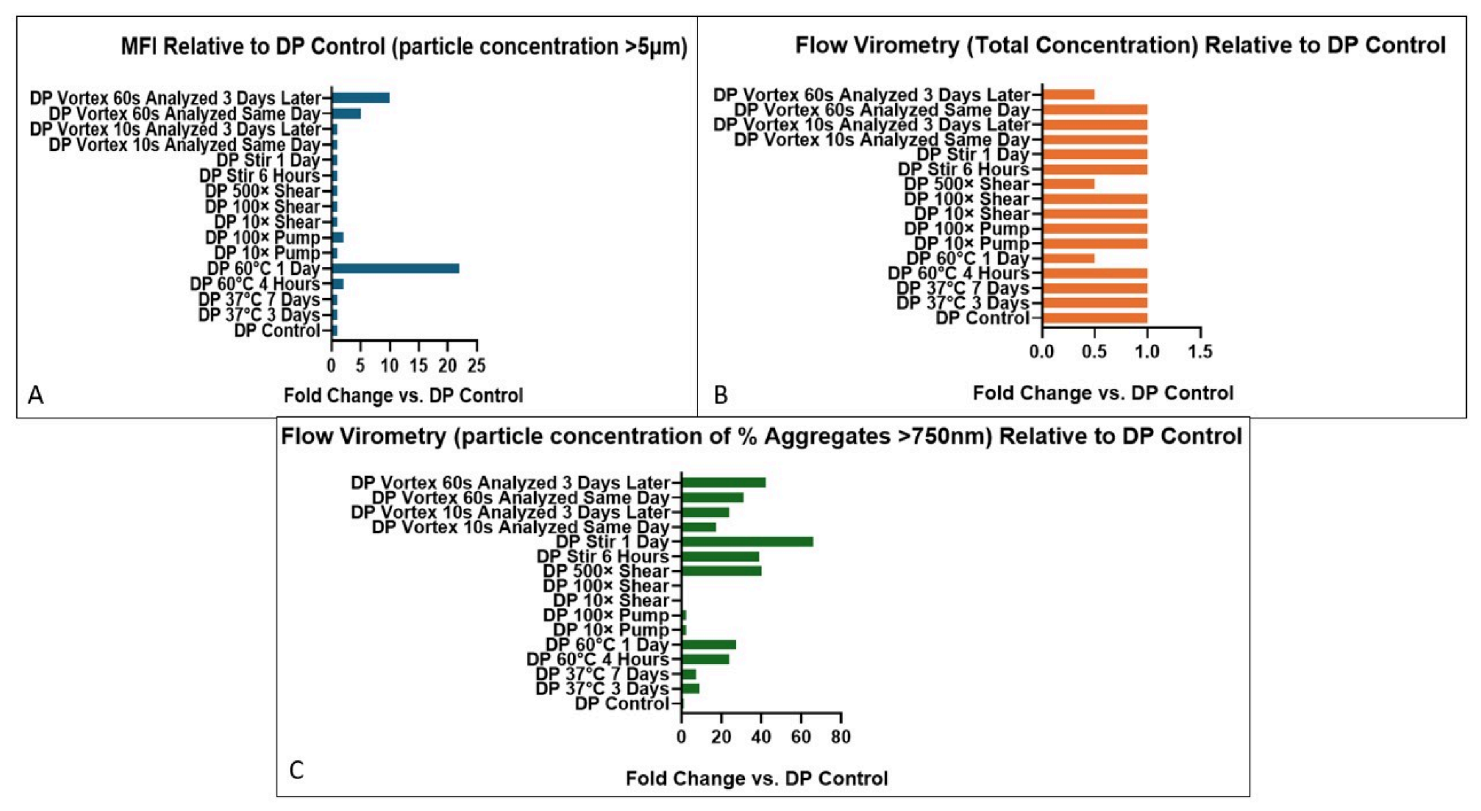
Summary of the results for DP measurements by MFI and flow virometry. (**A**) MFI particle concentration > 5 µm normalized to the DP control results. (**B**) Flow virometry total particle concentration normalized to the DP control results. (**C**) Flow virometry % aggregates > 750 nm normalized to the DP control results.

**Figure 8:**
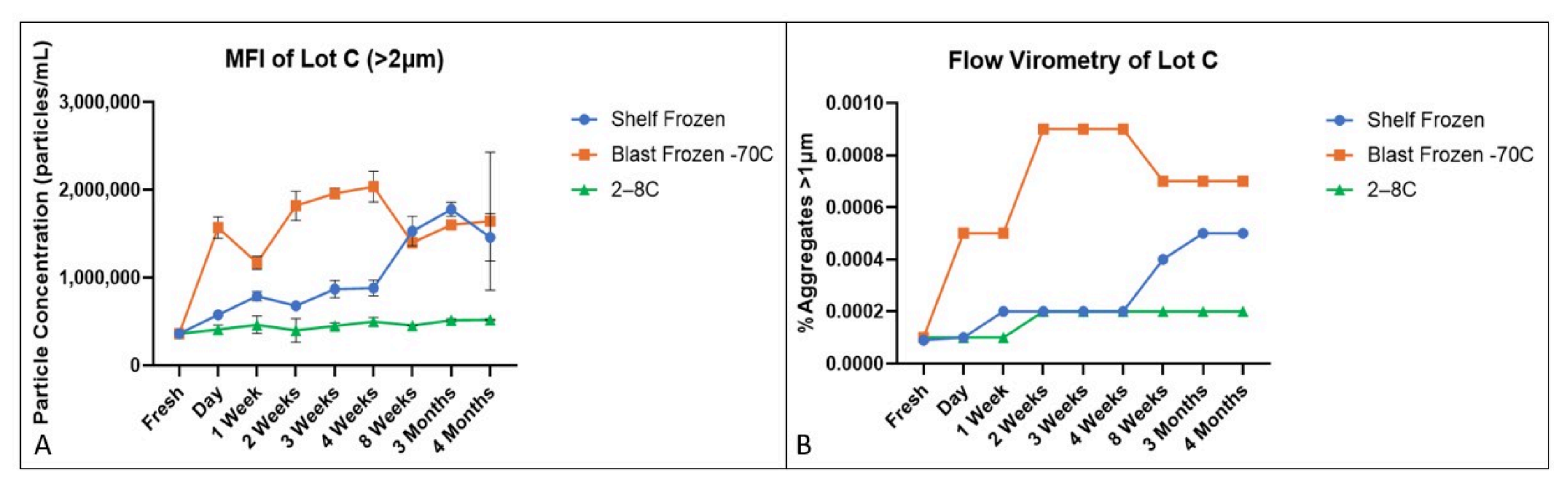
MFI, total particle concentration, and flow virometry of %aggregates > 1 µm for DS Lot C. (**A**) MFI results of Lot C stored at 2–8 °C and at −70 °C with and without blast freezing for 4 months. (**B**) Flow virometry results of Lot C stored at 2−8 °C and at −70 °C with and without blast freezing for 4 months.

**Figure 9:**
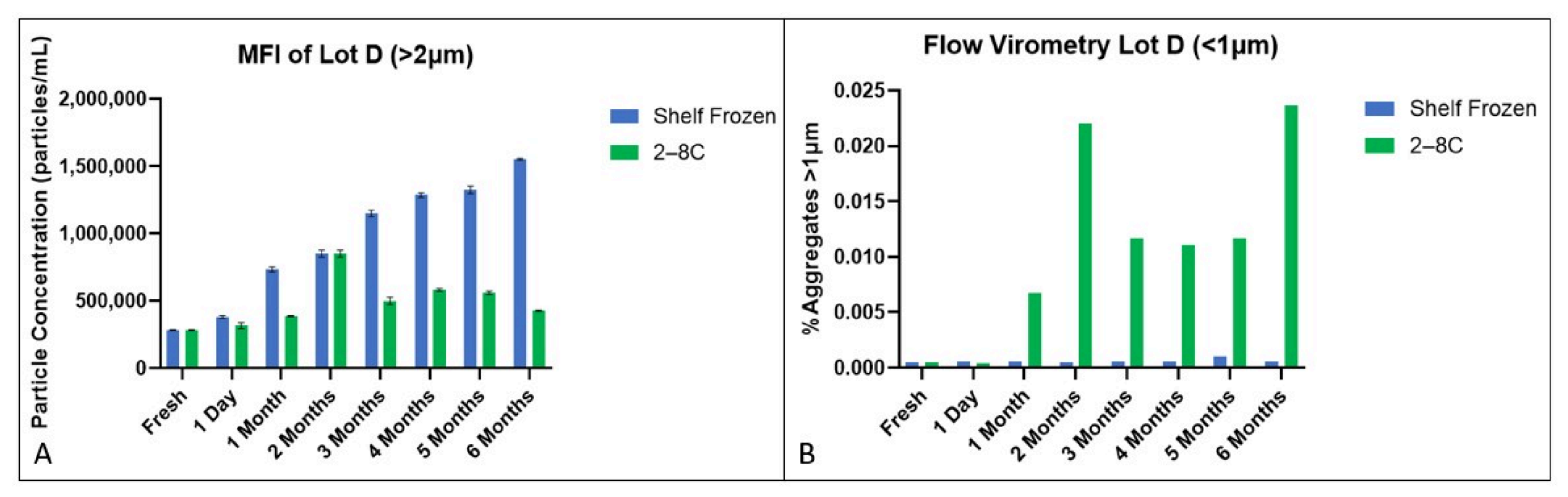
MFI, total particle concentration >2 µm, and flow virometry of %aggregates >1 µm for DS Lot D. (**A**) MFI results of Lot D stored at 2–8 °C and −70 °C for 6 months. (**B**) Flow virometry results of Lot D stored at 2–8 °C and −70 °C for 6 months.

**Figure 10:**
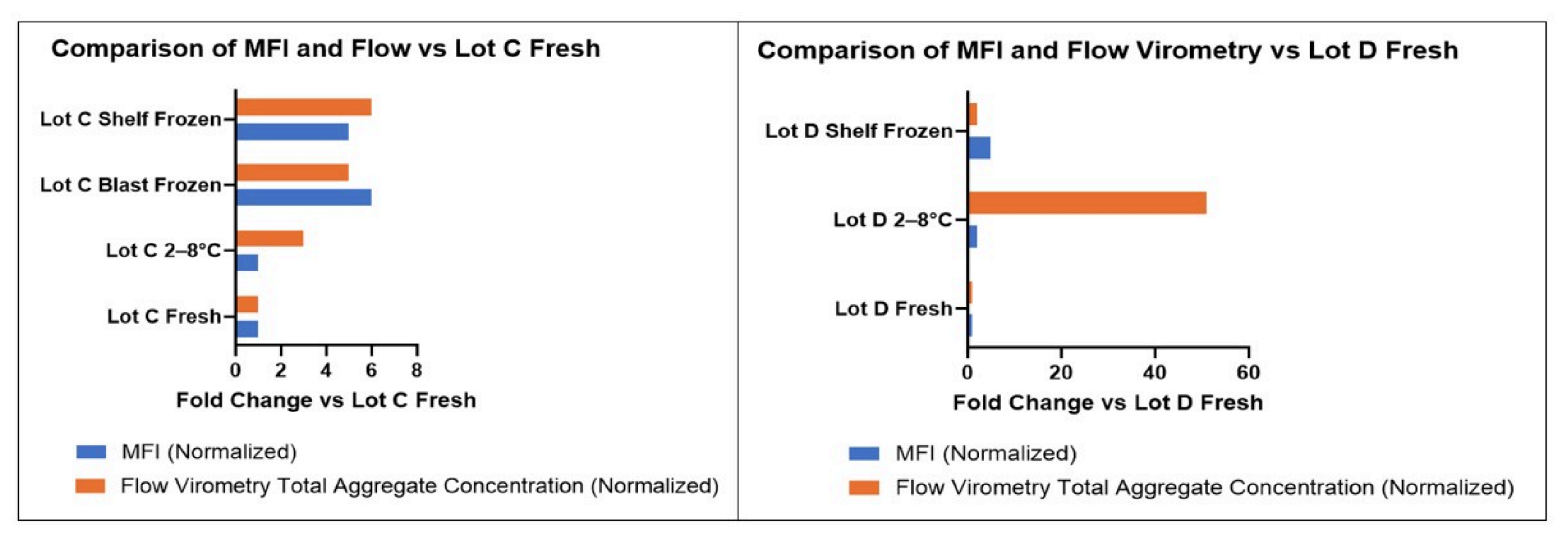
Summary of the results of thermal stability measurements by MFI and flow virometry for DS Lots C and D. Comparison of the MFI and flow virometry results. The data is normalized to the results of the fresh Lot C and Lot D.I I.

**Figure 11:**
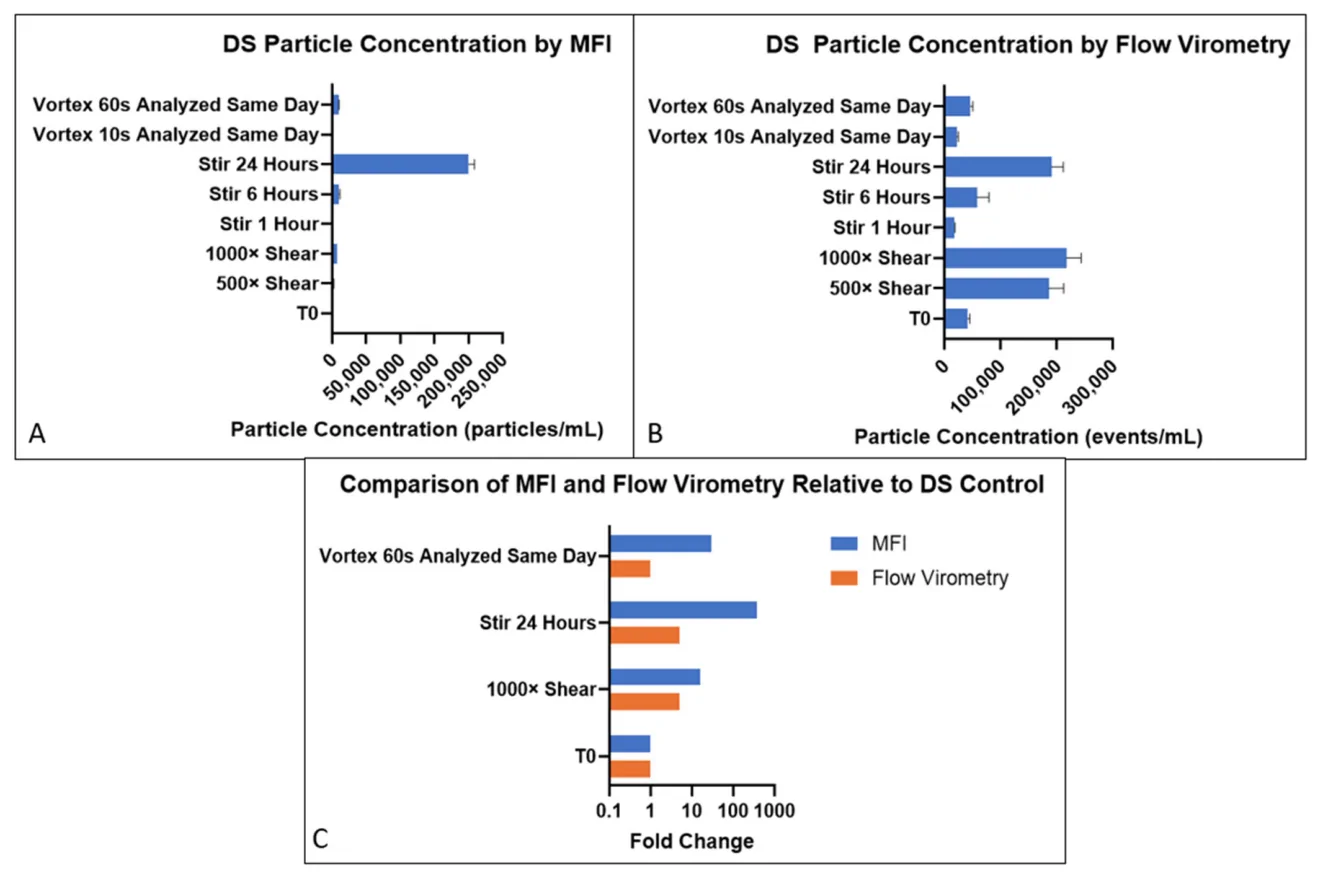
(**A**) MFI and total particle concentration > 1 µm of coxsackievirus A21 DS. Comparison of total particle concentration of DS after applying multiple stress conditions. (**B**) Flow virometry of coxsackievirus A21 DS. Comparison of total particle concentration of DS after applying multiple stress conditions. (**C**) Summary of the results for coxsackievirus A21 DS measurements by MFI and flow virometry. Comparison of MFI and flow virometry for coxsackievirus DS. The data is normalized to the results for the DS control.

## Data Availability

The data presented in this study are available upon request from the corresponding authors due to privacy restrictions.

## Supplementary Materials

The following supporting information can be downloaded at: https://www.mdpi.com/article/10.3390/vaccines14100839/s1, Figure S1: MFI images of DS aggregates Lot B post-stirring for 26 h; Table S1: DS bead particle size increase; Figure S2: Particle size distribution of polystyrene bead mixtures.

## Abbreviations

The following abbreviations are used in this manuscript:

DLS	Dynamic light scattering
DP	Drug product
DS	Drug substance
HCMV	Human cytomegalovirus
MFI	Micro-flow imaging
NIEPs	Non-infectious enveloped particles
NTA	Nanoparticle tracking analysis
VLPs	Virus-like particles
SDS-PAGE	Sodium dodecyl sulfate–polyacrylamide gel electrophoresis
SEC	Size-exclusion chromatography
TEM	Transmission electron microscopy
