# Supplementary material for: Micro-Flow Imaging and Flow Virometry as Preferred Methods to Monitor Aggregate Formation in the Submicron and Subvisible Size Range for Two Early-Phase Vaccine Candidates
Source: Vaccines (Basel). 2026 Sep 23;14(10):839. doi: 10.3390/vaccines14100839 (PMC13709367; doi:10.3390/vaccines14100839)
Supplement: Supplementary file 1 [file vaccines-14-00839-s001.zip › vaccines-4576648-supplementary.pdf]

Supporting Information

Figure S1. MFI Images of DS Aggregates Lot B Post Stirring for 26 Hours

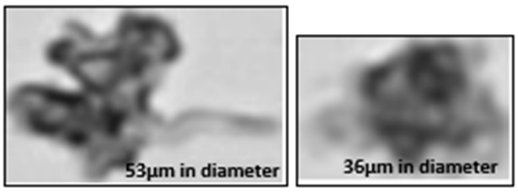

Polystyrene Bead Study

The DLS method is routinely used to monitor aggregation in vaccine samples due to its enhanced sensitivity for larger particles. The inability to significantly detect aggregation in HCMV samples prompted a study of polystyrene beads (see Table 2). Beads of 20 nm, 100 nm, 200 nm, 1 µm, and 5 µm sizes were mixed at various concentrations in order to mimic the particle sizes in HCMV samples. Particle size distribution of polystyrene bead mixtures was shown in Figure 4. Particle sizes in HCMV DS samples have measured from approximately 200 nm to 100 µm in diameter. To mimic the HCMV particle size range, beads were mixed in a 1:10 size ratio (20 nm mixed with 200 nm and 100 nm mixed with 1 µm). Because the virus measures 200 nm by DLS and 5 µm particles have been measured in DS samples by MFI, beads of 200 nm size were also mixed with 5 µm beads for a more relevant mock-up of the actual HCMV sample size range.

It was determined that as the particle with the highest concentration in the sample (primary particle) increases in size, the Z-ave of the sample is less affected by the larger, least concentrated particles (aggregates) in the sample. An increase in aggregate particle concentration is needed to significantly increase the average size. Therefore, by DLS the aggregate particle concentration in comparison to the primary particle concentration is too low in HCMV samples to significantly

increase the average particle size. According to the Rayleigh theory, particles scatter an equal amount of light in all directions proportional to the 6th power of the diameter [16]. As particle size increases Brownian motion also decreases. Light scattering becomes more dependent on scattering angle and less light is scattered in the back direction. DLS measures backscattering at an angle of 173° to avoid multiple scattering and large dust particles. Therefore, as the primary particle increases in size, the ratio of primary particle to aggregates plays a significant factor in aggregation detection by DLS. A 5-µm bead would have less Brownian motion and contribute less backscattering than a 200-nm bead. In HCMV DS samples, almost all of the light scattering comes from the 200 nm primary particles, which overpowers the weaker light scattering signal of larger, less abundant aggregated particles.

Table S1. DS Bead Particle Size Increase

Bead Mixture	Particle Ratio	Primary Particle Z-Ave (d.nm)	Bead Mixture Z-Ave (d.nm)	Size Increase of Bead Mixture (%)	% Mass of Aggregate
20 nm / 200 nm	100,000:1	22	71	105	200 nm = 0.001%
100 nm / 1 µm	100,000:1	104	104	0	1 µm = 0.001%
	10,000:1		121	17	1 µm = 0.001%
200 nm / 5 µm	10,000:1	203	210	3	5 µm = 0.010%
	4,000:1		225	10	5 µm = 0.025%
	1,000:1		1388	584	5 µm = 0.100%

Figure S2. Particle Size Distribution of Polystyrene Bead Mixtures

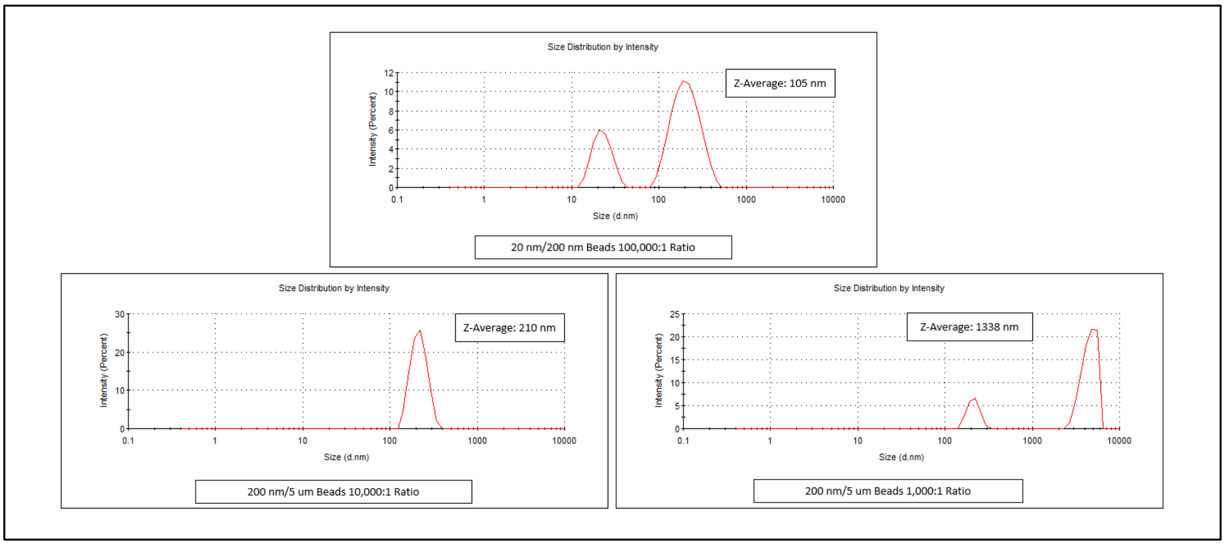
Size Distribution by Intensity
Z-Average: 105 nm
Intensity (Percent)
Size (d.nm)
20 nm/200 nm Beads 100,000:1 Ratio
Size Distribution by Intensity
Z-Average: 210 nm
Intensity (Percent)
Size (d.nm)
200 nm/5 um Beads 10,000:1 Ratio
Size Distribution by Intensity
Z-Average: 1338 nm
Intensity (Percent)
Size (d.nm)
200 nm/5 um Beads 1,000:1 Ratio
